# Renal Infiltration as a Primary Presentation of Burkitt Lymphoma Secondary to Systemic Lupus Erythematosus: A Rarity Unto a Rarity

**DOI:** 10.7759/cureus.10512

**Published:** 2020-09-17

**Authors:** Yusra Irshad, Ezza Fatima Tariq, Hajra Asif, Muhammad M Anwar, Usman A Khan

**Affiliations:** 1 Internal Medicine, Kulsoom International Hospital, Islamabad, PAK; 2 Internal Medicine, Nishtar Medical University and Hospital, Multan, PAK; 3 Nephrology, Oklahoma University Health Sciences Center, Oklahoma City, USA; 4 Internal Medicine, Quaid-e-Azam Medical College, Bahwalpur, PAK; 5 Biochemistry, King Edward Medical University (KEMU)/Mayo Hospital, Lahore, PAK; 6 Internal Medicine and Nephrology, University of Oklahoma Health Sciences Center, Oklahoma City, USA

**Keywords:** burkitt lymphoma, renal infiltration, acute kidney injury, systemic lupus erythematosus

## Abstract

Burkitt lymphoma (BL) is a highly aggressive non-Hodgkin B-cell lymphoma characterized by the translocation and deregulation of the MYC (MyeloCytomatosis) gene on chromosome 8. Three distinct clinical forms of BL are recognized: endemic (African), sporadic (non-endemic), and immunodeficiency-associated. Bilateral renal infiltration leading to acute kidney injury (AKI) is a rare initial presentation of BL. Diagnosis is usually made after evaluating the histology and immunophenotyping of the affected tissue.

We report a case of a 46-year-old male who presented with symptoms of AKI resulting from infiltrative disease, a primary presentation of lymphoma. The patient was a known case of systemic lupus erythematosus (SLE) for the last five years and was referred to the nephrology department due to acute elevation in creatinine, from 0.8 mg/dL to 3.57 mg/dL. On physical examination, there was no lymphadenopathy. Nephrology and SLE workup revealed low complement protein levels and absolute neutrophils, lymphocytes, and metamyelocytes. Renal ultrasound (USG) showed both kidneys with symmetric and edematous appearance. Biopsy affirmed high-grade B-cell lymphoma, positive for BCL-6 (B-cell leukemia/lymphoma) and CD-10 (cell surface marker) and negative for BCL-2 (B-cell leukemia/lymphoma). PET (positron emission tomography) scan showed extensive hypermetabolic lymphadenopathy in multiple areas. The patient was started on chemotherapy and on continuous renal replacement therapy. He improved clinically, and his creatinine lowered down to 0.8 mg/dL. Repeat USG showed decreased edematous appearance of both kidneys.

Primary renal infiltration by BL is a rare presentation in adults. Prompt renal biopsy will change the course of treatment and can affect the prognosis. It is thoroughly advised to keep this malignancy in mind when making a diagnosis for AKI.

## Introduction

Burkitt lymphoma (BL) is one of the extremely aggressive non-Hodgkin B-cell lymphoma. The pathogenesis involves translocation and dysregulation of the MYC (MyeloCytomatosis) gene on chromosome 8, which is an oncogene (8q24) and located on three other lg (immunoglobulin) genes: t(8;14), t(2;8), or t(8;22) [1-3]. It is a common pediatric tumor and encompasses 30% of non-endemic pediatric lymphoma with less than 1% of adult non-Hodgkin lymphoma. BL is generally divided into three forms on the basis of epidemiological and diagnostic tenacities; endemic (African), Sporadic (non-endemic), and immunodeficiency-associated [1]. Diagnosis is based on the amalgamation of clinical, histological, and immunophenotypical findings [2].

It has been reported in the literature [3] that there is 30-50% of renal involvement seen in the autopsies of non-Hodgkin lymphoma patients. Acute kidney injury (AKI) secondary to lymphoid cell infiltration is extremely rare, only 1% involvement is seen in acute leukemia, and in chronic leukemia and lymphoma, it is even less than 1% [4]. Renal failure seen in patients of non-Hodgkin lymphoma could be due to acute tumor lysis syndrome, urinary obstruction, or urate nephropathy [3-6], but diffuse bilateral kidney infiltration as a cause of AKI is a rare cause. Only a few cases have been reported in the literature [4-10].

## Case presentation

A 46-year-old male with a past medical history of systemic lupus erythematosus (SLE) for the last five years presented to his primary care physician for a routine follow-up. At the time of presentation, the patient had oliguria, but on physical examination, he had mild pallor and 1+ pedal edema with no rash and no lymphadenopathy. His laboratory results showed creatinine of 3.57 mg/dL (normal range: 0.70-1.30 mg/dL), which was an elevation from 0.80 mg/dL (one month back). He also had low complement levels (C3: 66 mg/dL [normal range: 100-233 mg/dL] and C4: 17.7 mg/dL [normal range: 14-48 mg/dL]). A working diagnosis of lupus nephritis was made, and the patient was transferred to our hospital for higher level of care.

**Figure 1:**
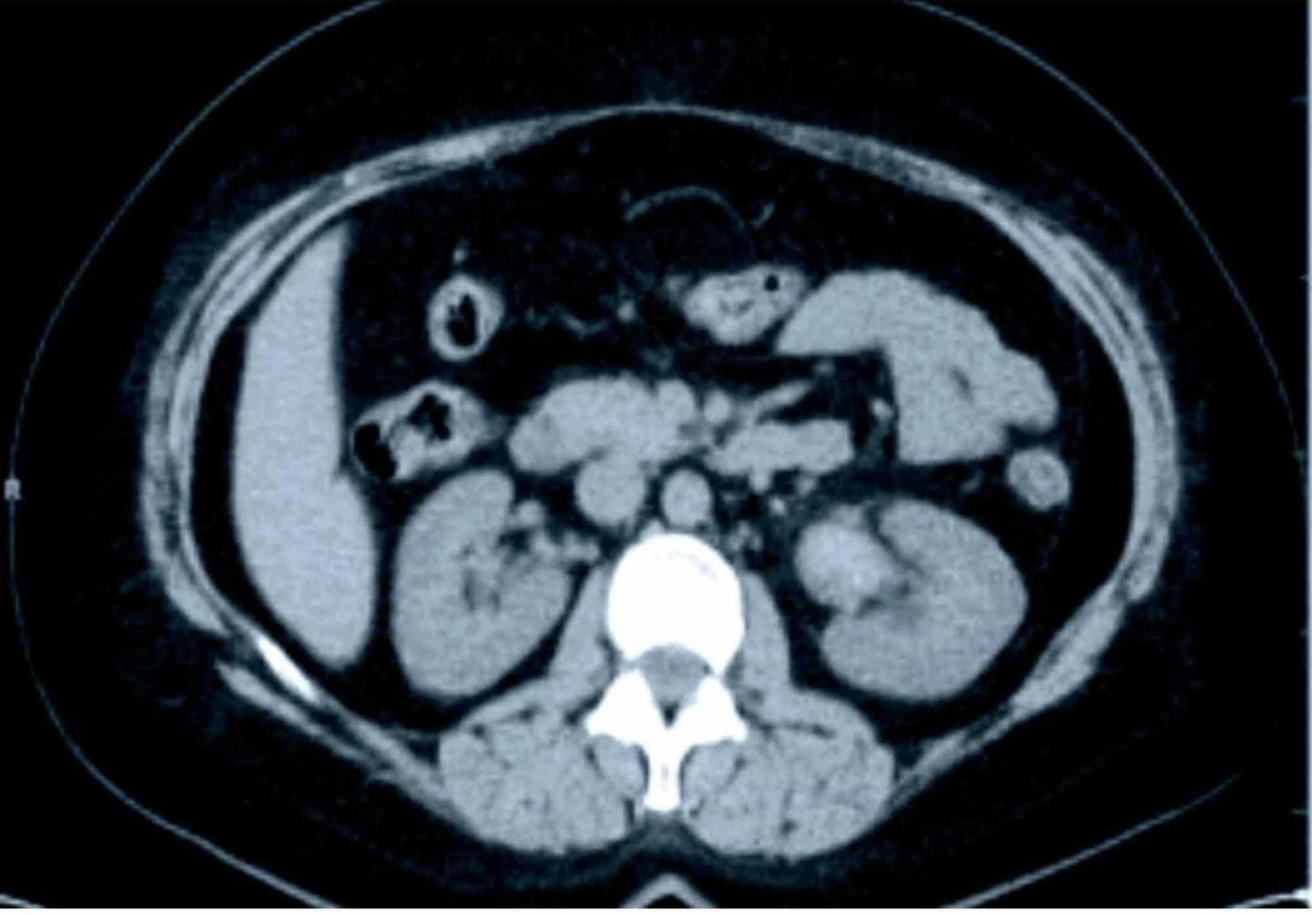
CT scan of the abdomen showing bilateral nephromegaly.

The patient had oliguria for the last four days but denied any hematuria, dysuria, increased urinary frequency, any new joint pain, rash, illicit drug use, fever, or swelling. AKI workup was ordered. CBC (complete blood count) revealed hemoglobin of 10.5 g/dL (normal range [NR]: 14-18 g/dL), WBC (white blood cell) count of 22.9 x 10^3^/microL (NR: 4,000-11,000/microL) with absolute band neutrophils of 3,435/microL (normal percentage [NP] in blood: 0-5%), absolute lymphocytes of 7,099/microL (NP: 30-45%), absolute metamyelocytes of 1,145/microL (not present in blood), absolute monocytes of 1,603/microL (NP: 0-6%), absolute neutrophils of 9,160/microL (NP: 50-70%), and platelets of 107 x 10^3^/microL (NR: 150,000-450,000/microL), which alarmed us for hematological malignancy. Routine urine examination was unremarkable with urine spot creatinine protein ratio of 1.2 (normal ratio less than 0.2), which was his baseline. HIV (human immunodeficiency virus) serology was negative. Renal Ultrasound and CT of the abdomen/pelvis (Figure 1) showed bilateral nephromegaly, with the right kidney measuring 14.8 cm (normal range: 11-13 cm) and the left kidney measuring 13.1 cm (normal range: 11-13 cm) with symmetric and edematous appearance to renal parenchyma bilaterally. The patient’s creatinine further elevated to 4.5 mg/dL the subsequent day, with BUN (blood urea nitrogen) of 40 mg/dL (normal range: 8-20 mg/dL).

**Figure 2:**
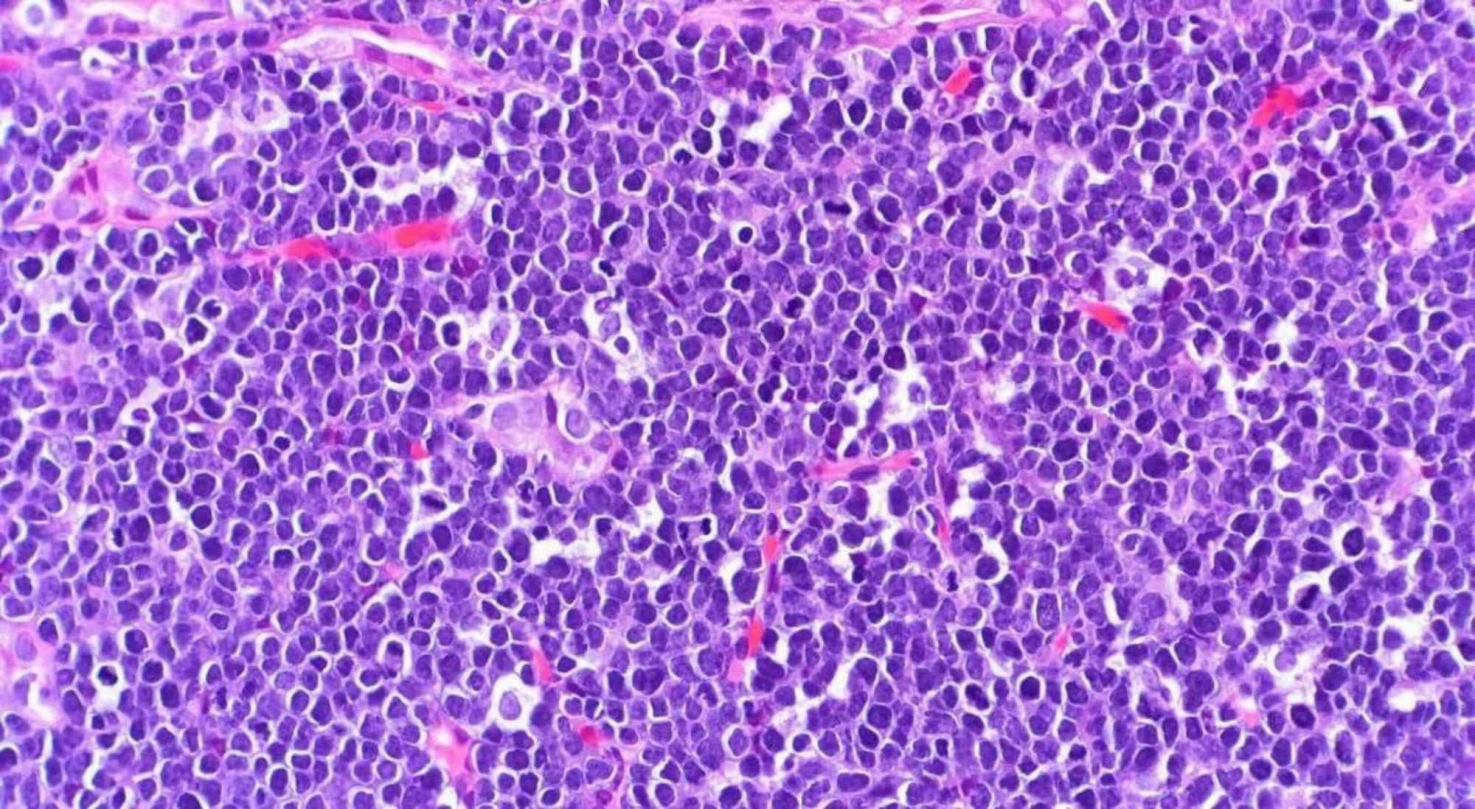
Renal tissue invaded by lymphoid cells.

Renal biopsy was performed, which showed high-grade B-cell (type of cell that produces antibodies) lymphoma, positive for BCL-6 (Burkitt cell leukemia/lymphoma) and CD-10 (weak) (cluster differentiation) and negative for BCL-2. The Ki-67 (monoclonal antibody) index was greater than 90%. The tentative diagnosis of high-grade lymphoma, BL (African) with MYC, and BCL-6 rearrangements was later confirmed by FISH (fluorescent in situ hybridization). Figure 2 shows renal tissue invaded by lymphoid cells.

**Figure 3:**
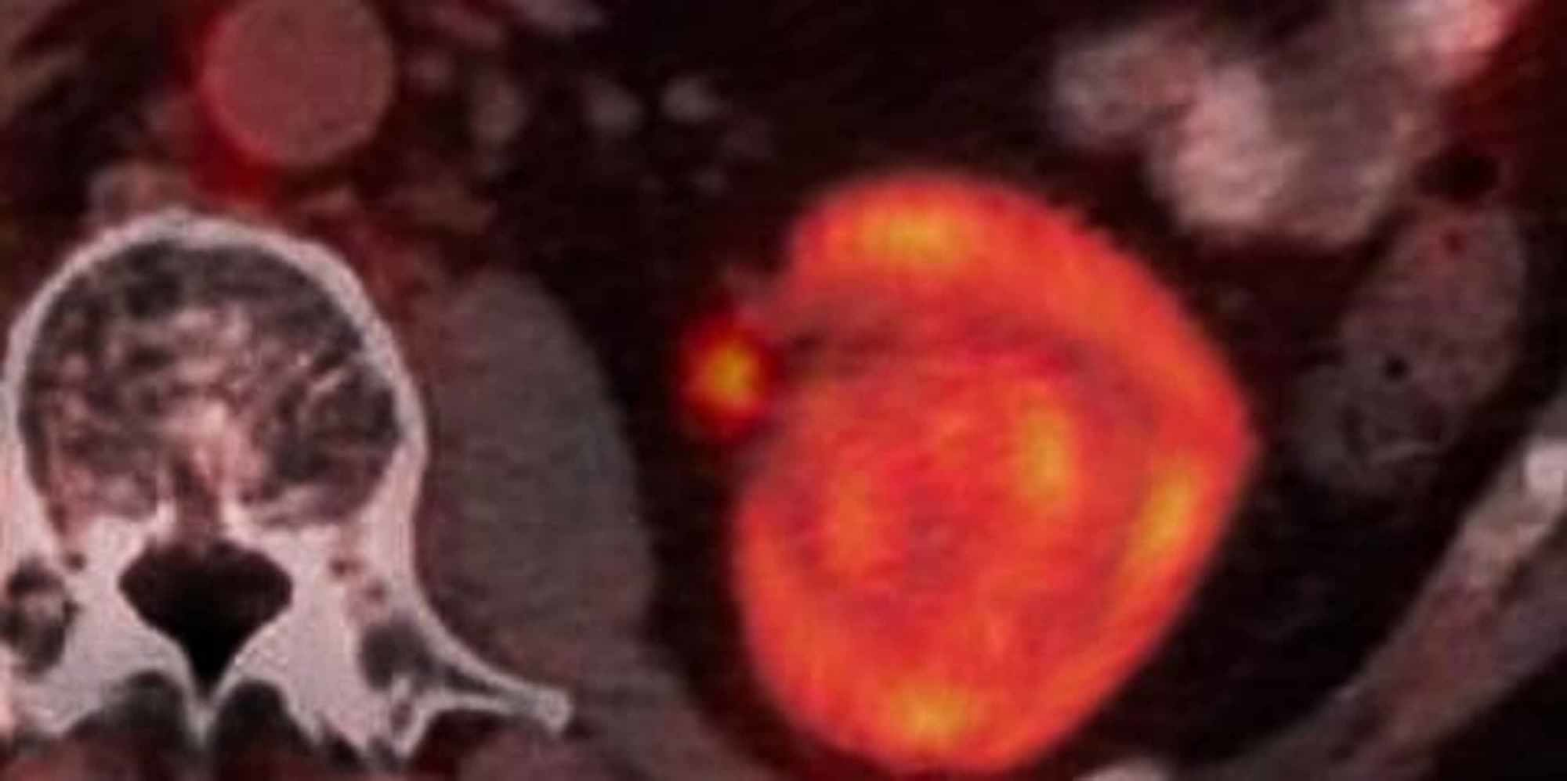
PET scan with increased FDG uptake in the right kidney. PET, positron emission tomography; FDG, fluorodeoxyglucose

The patient’s PET (positron emission tomography) scan (10/11) (Figure 3) showed extensive hypermetabolic lymphadenopathy in the cervical, mediastinal, subpectoral, axillary, abdominal, and pelvic regions consistent with BL. There was also diffuse hypermetabolic parenchymal disease in the kidneys and spleen. The patient was started on EDOCH (etoposide, doxorubicin, vincristine, cyclophosphamide, dexamethasone) chemotherapy regimen.

He developed severe acute tumor lysis syndrome after the first day of chemotherapy and was therefore started on continuous renal replacement therapy and later transitioned to intermittent hemodialysis on day 4. He stayed at the hospital for nine days and started to maintain his urine output after the second week of chemotherapy. The patient was taken off hemodialysis subsequently, and the tunneled dialysis catheter was removed. He was routinely followed up every two weeks. Creatinine trended down to 0.8 in the next two months. The patient’s repeat renal USG (six weeks post-chemotherapy) showed the right kidney measuring 12.5 cm in size and the left kidney measuring 11.8 cm in size.

## Discussion

BL is derived from germinal or post-germinal center B cells (Ig-producing cells). The development of BL is dependent on the constitutive expression of the MYC proto-oncogene located at chromosome 8q24, which encodes the MYC [1-3] protein transcription factor V. This transcription factor modulates the expression of target genes that regulate many cell processes including cell growth and division, immortalization, Warburg metabolism (modified cellular metabolism in cancer cells), and cell death by apoptosis. In 1958, an English surgeon, Burkitt, described the respective lymphoma as a highly aggressive non-Hodgkin B-cell lymphoma that resembles acute lymphoblastic leukemia subtype L3 (classification of the tumor according to the shape of cells and nucleus) [2,4,6,7]. It commonly affects male children between four and eight years of age, but it can occur at any age [1,4]. The highest incidence of BL is seen in developing countries (especially Equatorial Africa), but due to limited resources required for accurate diagnosis, the exact worldwide incidence is not known [1,2]. It is generally divided into three forms on the basis of epidemiological and diagnostic purposes: endemic (African), Sporadic (non-endemic), and immunodeficiency-associated. The endemic and sporadic clinical variants of BL differ geographically [1].

In comparison to Hodgkin lymphoma, renal involvement is commonly seen in non-Hodgkin lymphoma. but glomerular disease is prevalent in Hodgkin disease. Minimal change disease, a type of glomerulonephritis, has been reported in both types of lymphomas [5]. Impairment of renal function seen in patients could be due to multiple reasons, but obstructive uropathy is the most common one. It transpires when there is either tumor invasion/retroperitoneal fibrosis or direct compression of the urinary tract by affected retroperitoneal lymph nodes [3-5]. A life-threatening oncological emergency can occur after chemotherapy, which causes tumor lysis syndrome, resulting in abrupt renal failure [4,11]. In 2008, an international expert panel published evidence-based guidelines for the prevention and management of tumor lysis syndrome which placed ALL (acute lymphoblastic leukemia), stage III or IV BL, or early stage BL with serum LDH (lactate dehydrogenase) level two or more times the upper limit of normal at the highest risk (>6%) [11].

Renal infiltration by lymphoid cells as initial manifestation of acute renal failure (ARF) is a rare cause [6,7,9]. In the autopsy of non-Hodgkin lymphoma patients, the incidence of renal involvement was 30-50% (that was clinically silent with no symptoms), out of which only 0.9-23% developed renal insufficiency [5]. Seven cases of Burkitt lymphoma presenting as ARF [4-10] have been listed in the literature so far, whereas only one case had been reported where a patient with SLE [12] also developed BL.

Prognosis of lymphomas depends on the response to the chemotherapy. The early treatment with EDOCH chemotherapy regimen can quickly improve the renal function and ultimately prognosis [5]. Acute renal insufficiency with enlarged kidney size on the ultrasound and abnormal peripheral smear with myelocytes and blasts should raise suspicion for non-Hodgkin lymphoma. After ruling out other common causes of ARF, early renal biopsy should be considered in these patients provided that there are no lymph nodes in biopsy [9,10]. As early initiation of chemotherapy is prognostically important for the primary disease and renal failure.

## Conclusions

Bilateral renal infiltration by BL presenting as AKI is a rare primary presentation in adults and can be misdiagnosed easily. Primary renal infiltration as the first presenting symptom is uncommonly reported in the literature. Imaging may give a clue about the possibility of lymphomatous infiltration, and renal biopsy is the gold standard for diagnosis; early biopsy in this situation will change the course of the treatment, and prompt chemotherapy will improve renal functions, therefore decreasing mortality. The possibility of renal infiltrative lymphoma, a diagnosable and treatable condition, should be considered as a cause of AKI in patients with bilateral nephromegaly with no obvious explanation.
